# Safety and efficacy of amrubicin with primary prophylactic pegfilgrastim as second‐line chemotherapy in patients with small cell lung cancer

**DOI:** 10.1111/1759-7714.15140

**Published:** 2023-10-24

**Authors:** Motoki Sekikawa, Haruyasu Murakami, Meiko Morita, Kosei Doshita, Keita Miura, Hiroaki Kodama, Noboru Morikawa, Yuko Iida, Nobuaki Mamesaya, Haruki Kobayashi, Ryo Ko, Kazushige Wakuda, Akira Ono, Hirotsugu Kenmotsu, Tateaki Naito, Hirofumi Chiba, Toshiaki Takahashi

**Affiliations:** ^1^ Division of Thoracic Oncology Shizuoka Cancer Center Shizuoka Japan; ^2^ Department of Respiratory Medicine and Allergology Sapporo Medical University School of Medicine Sapporo Japan

**Keywords:** amrubicin, febrile neutropenia, pegfilgrastim, second‐line chemotherapy, small cell lung cancer

## Abstract

**Background:**

Amrubicin (AMR) regimens have shown efficacy as second‐line treatment in patients with small cell lung cancer (SCLC); however, adverse events such as febrile neutropenia (FN) sometimes preclude their use. Further, the safety and efficacy of AMR with primary prophylactic pegfilgrastim (P‐PEG) have not been sufficiently evaluated. In this study, we evaluated the safety and efficacy of AMR with or without P‐PEG as second‐line chemotherapy for SCLC.

**Methods:**

We retrospectively reviewed patients with SCLC who received AMR as second‐line chemotherapy at Shizuoka Cancer Center, between December 2014 and November 2021. Based on presence/absence of P‐PEG in their regimen, patients (*n* = 60) were divided into P‐PEG (*n* = 21) and non‐P‐PEG groups, and their clinical outcomes were evaluated.

**Results:**

Median of AMR treatment cycles was five (range: 1–39 cycles) in P‐PEG group and four (range: 1–15 cycles) in non‐P‐PEG group. The incidence of FN (4.8% vs. 30.8%; *p* = 0.02) and AMR dose reduction because of adverse events (4.8% vs. 25.6%; *p* = 0.08) were lower in the P‐PEG group than in the non‐P‐PEG group. The objective response rates were 52.4% and 30.8%, and median progression‐free and overall survival were 4.7 and 3.0 months, and 9.6 and 6.8 months, in the P‐PEG and non‐P‐PEG groups, respectively.

**Conclusions:**

AMR with P‐PEG as second‐line chemotherapy for SCLC reduced the incidence of FN at a maintained AMR dose intensity and was associated with favorable tumor responses and survival outcomes. P‐PEG should be considered for patients treated with AMR for SCLC including refractory relapsed SCLC.

## Graphical abstract

Amrubicin (AMR) in combination with primary prophylactic pegfilgrastim (P‐PEG) as second‐line chemotherapy for small cell lung cancer (SCLC) reduced the incidence of febrile neutropenia and was associated with favorable tumor responses. Survival outcomes such as progression‐free and overall survival tended to be better in AMR with P‐PEG compared to without P‐PEG. P‐PEG should be considered for patients treated with AMR for SCLC, especially those with refractory relapsed SCLC.

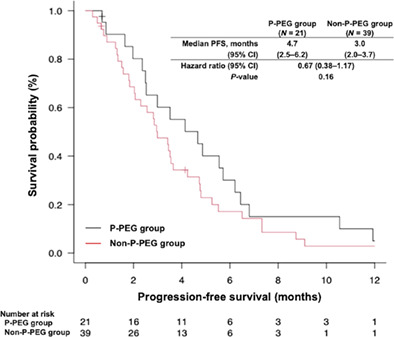

## INTRODUCTION

Lung cancer is one of the most common malignancies and the leading cause of death worldwide.^1^ Small cell lung cancer (SCLC) accounts for 13%–14% of all newly diagnosed lung cancers.^2^, ^3^ SCLC has a high frequency of distant metastasis at the time of diagnosis, and more than 70% of patients are diagnosed with extensive disease.^4^ Platinum‐based chemotherapy has long been used as the first‐line treatment for SCLC. In recent years, platinum doublet plus immune checkpoint inhibitors have been introduced for SCLC and have since improved the overall survival (OS).^5^, ^6^ However, most SCLC cases show recurrence; second‐line treatment options for SCLC include amrubicin (AMR),^7^, ^8^, ^9^, ^10^, ^11^ topotecan,^12^ combined chemotherapy with carboplatin and etoposide,^13^ and combined chemotherapy with cisplatin, etoposide, and irinotecan.^14^

AMR therapy has demonstrated favorable tumor responses and survival outcomes in patients with relapsed SCLC; however, this agent can cause hematological toxicity, particularly febrile neutropenia (FN), with an occurrence of 10%–27% reported in clinical trials.^7^, ^8^, ^9^, ^10^, ^11^ A systematic review of multiple cancer types reported that the primary prophylactic administration of granulocyte colony‐stimulating factor (G‐CSF) is associated with significantly better chemotherapy dose intensity and reduced mortality,^15^ with pegfilgrastim (PEG) being commonly used for primary prophylactic administration in recent years. Although dose intensity contributes to improved clinical outcomes in terms of first‐line chemotherapy for SCLC,^16^ the importance of dose intensity in second‐line chemotherapy with AMR has not been sufficiently evaluated. Therefore, in this study, we evaluated the safety and efficacy of AMR with or without primary prophylactic PEG (P‐PEG) as second‐line chemotherapy in patients with SCLC.

## METHODS

### Patients

We retrospectively collected data from patients with SCLC who received AMR at a dose of 40 mg/m^2^ as second‐line chemotherapy at Shizuoka Cancer Center between December 2014 and November 2021. The patients were grouped and evaluated separately as those either receiving or not receiving P‐PEG. The date for data cutoff was March 31, 2022. This study was approved by the Institutional Review Board of Shizuoka Cancer Center and conducted in accordance with the principles stated in the Ethical Guidelines for Medical and Biological Research Involving Human Subjects.^17^ An opt‐out method was adopted to obtain patient consent.

### Treatment

All patients received AMR every 3 weeks at a dose of 40 mg/m^2^ intravenously on days 1–3. The decision to administer P‐PEG was based on consultation between the attending physician and the patient. In the P‐PEG group, primary prophylactic PEG was administered subcutaneously after day 4 of AMR therapy. In the non‐P‐PEG group, G‐CSF was administered in cases where FN and/or grade 4 neutropenia developed. For patients in the non‐P‐PEG group who had FN or severe neutropenia in the previous course, prophylactic PEG was administered on day 4 of the second and subsequent courses, which was defined as secondary prophylactic PEG. The attending physician also decided whether to reduce the AMR dose depending on the adverse events that developed in the previous cycle. When dose reduction was performed, the dose was reduced stepwise to 35 mg/m^2^, and then to 30 mg/m^2^. Treatment was continued until disease progression was observed, unacceptable toxicity, and/or patient request.

### Evaluation

Clinical data such as age, sex, Eastern Cooperative Oncology Group performance status (PS), previous regimen, episode of FN prior to chemotherapy, and history of thoracic radiation therapy were collected from patient medical records. Both refractory and sensitive relapse were included in the study; refractory relapse was defined as no response to first‐line chemotherapy, disease progression while on first‐line chemotherapy, or disease progression within 90 days of completing first‐line chemotherapy after confirming a complete or partial response. Adverse events were graded using the Common Terminology Criteria for Adverse Events (version 5.0). Tumor response was assessed according to the Response Evaluation Criteria for Solid Tumors (version 1.1).^18^ Progression‐free survival (PFS) was defined as the time from the first day of AMR administration to the earlier date of disease progression or death. OS was defined as the time from the first day of AMR administration until death.

### Statistical analysis

Fisher's exact test was used to compare categorical data as follows: patient characteristics, AMR treatment delivery, adverse events, and tumor response. Survival estimation was performed using the Kaplan–Meier method, and subgroups were compared using the log‐rank test. We calculated the hazard ratio for PFS and OS using the Cox proportional hazards model. All statistical tests were two‐sided, and a *p*‐value less than 0.05 was considered to indicate statistical significance. All analyses were performed using EZR version 1.55 (Saitama Medical Center, Jichi Medical University, Saitama, Japan).^19^

## RESULTS

### Patient characteristics

A total of 60 patients were enrolled in the study: 21 in the P‐PEG group and 39 in the non‐P‐PEG group. The patient characteristics are summarized in Table 1. No significant differences in patient characteristics were observed between the two groups. The majority of patients in both groups had a PS of either 0 or 1, extensive disease, refractory relapse, and no episode of FN upon prior chemotherapy. All 60 patients were pretreated with platinum doublet chemotherapy; three patients in the P‐PEG and five patients in the non‐P‐PEG groups received immune checkpoint inhibitors in combination with platinum doublet chemotherapy.

**TABLE 1:** Patient characteristics.

	P‐PEG group (*N* = 21)		Non‐P‐PEG group (*N* = 39)		
Characteristic	*N*	%	*N*	%	*p‐*value
Age, years					
Median	64		68		
Range	46–79		53–76		
<71	18	85.7	29	74.4	0.51
≥71	3	14.3	10	25.6	
Sex					
Female	3	14.3	8	20.5	0.73
Male	18	85.7	31	79.5	
ECOG performance status					
0	8	38.1	7	18.0	0.18
1	11	52.4	29	74.4	
2	2	9.5	3	7.7	
Type of relapse					
Sensitive	3	14.3	4	10.3	0.69
Refractory	18	85.7	35	89.7	
Disease extent at initial diagnosis					
Limited disease	2	9.5	2	5.1	0.61
Extensive disease	19	90.5	37	94.9	
History of thoracic radiation therapy					
Yes	5	23.8	5	12.8	0.30
No	16	76.2	34	87.2	
Prior chemotherapy					
Cisplatin‐containing	6	28.6	21	53.8	0.10
Carboplatin‐containing	15	71.4	18	46.2	0.10
Irinotecan‐containing	6	28.6	16	41.0	0.41
Etoposide‐containing	15	71.4	23	59.0	0.41
ICI‐containing	3	14.3	5	12.8	1.00
Episode of FN at prior chemotherapy					
Yes	1	4.8	2	5.1	1.00
No	20	95.2	37	94.9	
Response to prior chemotherapy					
Complete response	1	4.8	0	0.0	0.50
Partial response	19	90.5	34	87.2	
Stable disease	0	0.0	3	7.7	
Progressive disease	1	4.8	2	5.1	

Abbreviations: ECOG, Eastern Cooperative Oncology Group; FN, febrile neutropenia; ICI, immune checkpoint inhibitor; P‐PEG, primary prophylactic pegfilgrastim.

### Treatment delivery and safety

The administration regimen of AMR is summarized in Table 2. The median number of AMR treatment cycles was five (range: 1–39 cycles) in the P‐PEG group and four (range: 1–15 cycles) in the non‐P‐PEG group (*p* = 0.10). AMR dose reduction tended to be less frequent in the P‐PEG group compared with that in the non‐P‐PEG group (4.8% vs. 25.6%; *p* = 0.08). The reasons for dose reduction were grade 4 neutropenia (*N* = 1) in the P‐PEG group, and FN (*N* = 7) and grade 4 neutropenia (*N* = 3) in the non‐P‐PEG group. Treatment was discontinued in one patient (grade 3 pneumonia) in the P‐PEG group and three patients (grade 3 FN) in the non‐P‐PEG group, because of toxicity. Among the 32 patients who received more than two cycles of AMR in the non‐P‐PEG group, seven received secondary prophylactic PEG.

**TABLE 2:** Treatment delivery of amrubicin.

	P‐PEG group (*N* = 21)		Non‐P‐PEG group (*N* = 39)		
	*N*	%	*N*	%	*p*‐value
Number of treatment cycles					
Median	5		4		0.10
Range	1–39		1–15		
Dose reduction	1	4.8	10	25.6	0.08
Febrile neutropenia	0	0.0	7	17.9	0.09
Grade 4 neutropenia	1	4.8	3	7.7	1.00
Discontinuation	21	100	38	97.4	1.00
Progressive disease	19	90.5	35	89.7	1.00
Toxicity	1	4.8	3	7.7	1.00
Patient request	1	4.8	0	0.0	0.35
Secondary prophylactic PEG	‐	‐	7	17.9	‐

Abbreviations: P‐PEG, primary prophylactic pegfilgrastim; PEG, pegfilgrastim.

Grade 3 or higher adverse events in the patients are summarized in Table 3. Hematological toxicity was commonly observed in both groups. The incidence of grade 3 or higher neutropenia (19.0% vs. 74.4%; *p* < 0.01), leukopenia (19.0% vs. 51.3%; *p* = 0.03), and FN (4.8% vs. 30.8%; *p* = 0.02) was significantly lower in the P‐PEG group than in the non‐P‐PEG group. The incidence of grade 3 or higher adverse events was significantly decreased in the P‐PEG group compared with that in the non‐P‐PEG group.

**TABLE 3:** Grade 3 or higher adverse events.

Adverse event (CTCAE v 5.0)	P‐PEG group (*N* = 21)		Non‐P‐PEG group (*N* = 39)		*p*‐value
	≥grade 3		≥grade 3		
	*N*	%	*N*	%	
Neutropenia	4	19.0	29	74.4	<0.01
Leukopenia	4	19.0	20	51.3	0.03
Anemia	1	4.8	4	10.3	0.65
Thrombocytopenia	2	9.5	9	23.1	0.30
Febrile neutropenia	1	4.8	12	30.8	0.02
Infection	0	0.0	2	5.1	0.54
Pneumonia	1	4.8	0	0.0	0.35
Anorexia	0	0.0	3	7.7	0.55
Diarrhea	0	0.0	1	2.6	1.00
Constipation	0	0.0	1	2.6	1.00
Hyponatremia	0	0.0	1	2.6	1.00

Abbreviations: CTCAE, common terminology criteria for adverse events; P‐PEG, primary prophylactic pegfilgrastim.

### Efficacy

**FIGURE 1:**
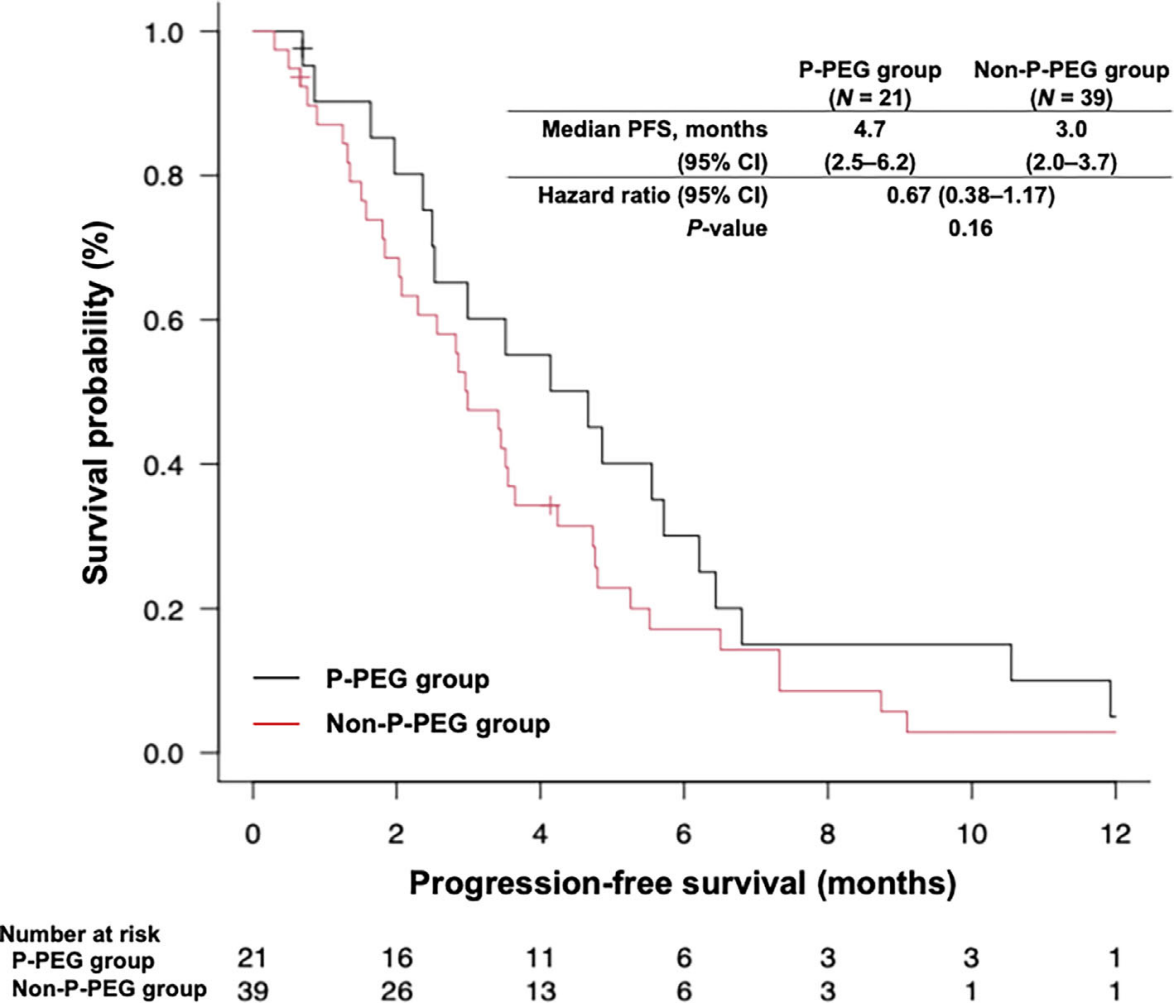
Kaplan–Meier analysis of PFS in P‐PEG and non‐P‐PEG groups. CI, confidence interval; PFS, progression‐free survival; P‐PEG, primary prophylactic pegfilgrastim.

**FIGURE 2:**
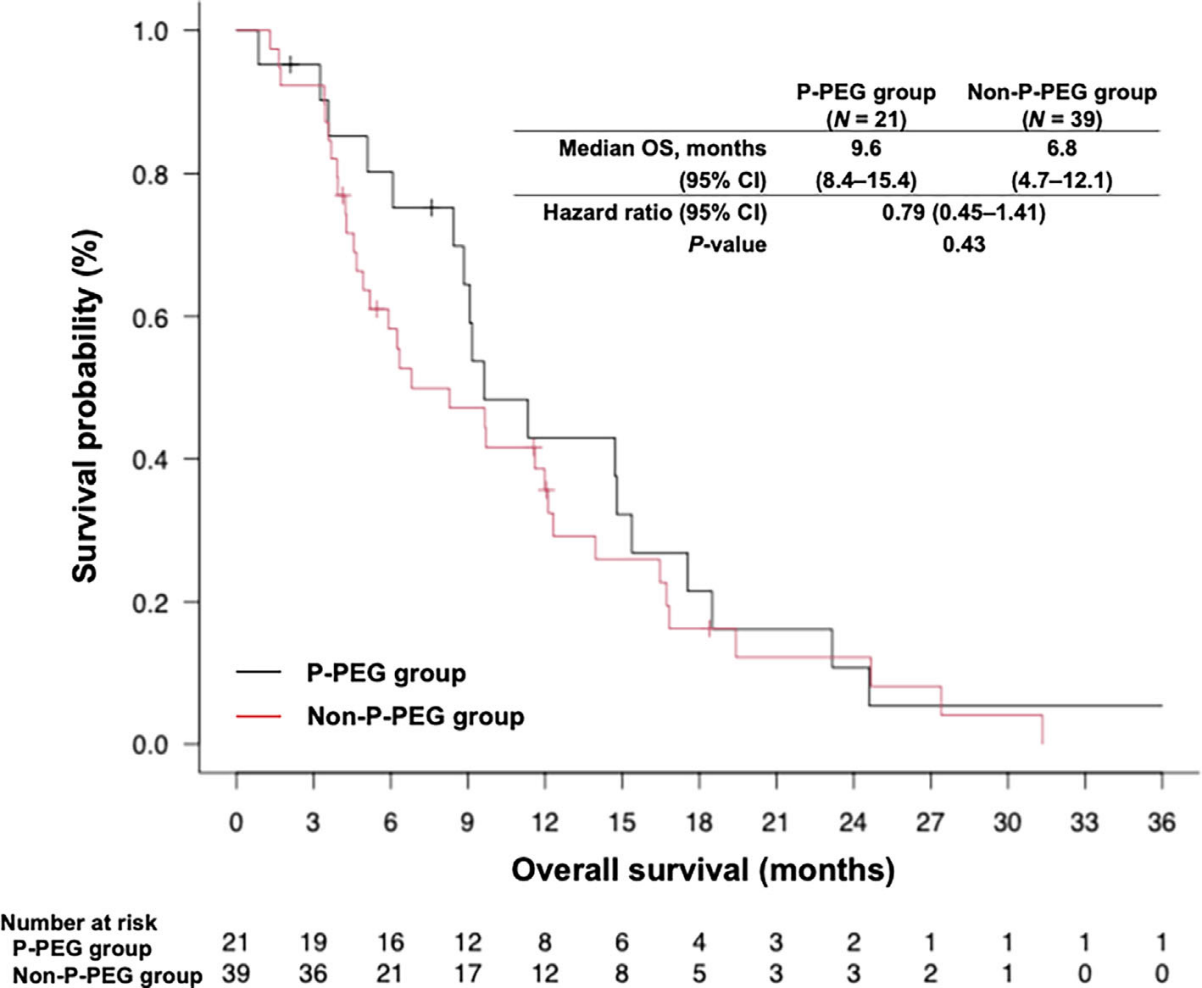
Kaplan–Meier analysis of OS in P‐PEG and non‐P‐PEG groups. CI, confidence interval; OS, overall survival; PFS, progression‐free survival; P‐PEG, primary prophylactic pegfilgrastim.

The objective response rate (ORR) was 52.4% and 30.8% in the P‐PEG and non‐P‐PEG groups, respectively (Table 4). No significant difference in the ORR was observed between the two groups (*p* = 0.16). The median PFS was 4.7 months (95% confidence interval [CI]: 2.5–6.2 months) in the P‐PEG group and 3.0 months (95% CI: 2.0–3.7 months) in the non‐P‐PEG group (hazard ratio for death or disease progression, 0.67; 95% CI: 0.38–1.17; *p* = 0.16) (Figure 1). The median OS was 9.6 months (95% CI: 8.4–15.4 months) in the P‐PEG group and 6.8 months (95% CI: 4.7–12.1 months) in the non‐P‐PEG group (hazard ratio for death, 0.79; 95% CI: 0.45–1.41; *p* = 0.43) (Figure 2).

**TABLE 4:** Response to amrubicin.

Tumor response	P‐PEG group (*N* = 21)		Non‐P‐PEG group (*N* = 39)		
	*N*	%	*N*	%	*p*‐value
CR	0	0.0	0	0.0	
PR	11	52.4	12	30.8	
SD	4	19.0	13	33.3	
PD	4	19.0	13	33.3	
NE	2	9.5	1	2.6	
ORR (CR + PR)	11	52.4	12	30.8	0.16

Abbreviations: CR, complete response; NE, not evaluated; ORR, objective response rate; PD, progressive disease; P‐PEG, primary prophylactic pegfilgrastim; PR, partial response; SD, stable disease.

In a subset analysis of 53 patients with refractory relapsed SCLC (18 in the P‐PEG group and 35 in the non‐P‐PEG group), the ORR was 44.4% and 26.6% in the P‐PEG and non‐P‐PEG groups, respectively. The median PFS values were 3.5 months (95% CI: 2.0–5.6 months) in the P‐PEG group and 3.0 months (95% CI: 2.0–3.6 months) in the non‐P‐PEG group (hazard ratio for death or disease progression, 0.73; 95% CI: 0.40–1.33; *p* = 0.29). The median OS was 9.2 months (95% CI: 5.1–14.8 months) in the P‐PEG group and 6.8 months (95% CI: 4.6–12.3 months) in the non‐P‐PEG group (hazard ratio for death, 0.89; 95% CI: 0.48–1.66; *p* = 0.73).

## DISCUSSION

In this study, we evaluated the safety and efficacy of AMR with or without P‐PEG as second‐line chemotherapy in 60 patients with SCLC. Among them, 21 (35.0%) received AMR therapy with P‐PEG. Although there were no significant differences in patient characteristics between the groups, the incidence of FN and AMR dose reduction because of adverse events was lower in the P‐PEG group than in the non‐P‐PEG group. In a previous prospective study, the incidence of FN and AMR dose reduction in patients with SCLC was 27% and 38%, respectively, similar to that in patients in the non‐P‐PEG group in the present study.^8^ According to the guidelines of the American Society of Clinical Oncology, the prophylactic use of hematopoietic colony‐stimulating factors to reduce the risk of FN is warranted when the risk of FN is approximately 20% or higher and no other equally effective and safe regimen that does not require hematopoietic colony‐stimulating factors is available.^20^ Although several other treatment options for sensitive relapsed SCLC are available apart from AMR, including topotecan, combined chemotherapy with carboplatin and etoposide and combined chemotherapy with cisplatin, etoposide, and irinotecan, AMR is the only recommended treatment regimen for refractory relapsed SCLC. Therefore, AMR in combination with P‐PEG is a reasonable treatment option for patients with refractory relapsed SCLC.

In a recent retrospective study, the real‐world incidence of FN in patients with thoracic malignancies, including SCLC treated with AMR, was 30%, and the OS of patients with FN was significantly shorter than that of patients without.^21^ In terms of efficacy, AMR with P‐PEG as second‐line chemotherapy for SCLC, including refractory relapsed SCLC, was associated with a favorable tumor response and survival outcome in the present study. Dose reduction and the discontinuation of AMR because of adverse events, such as severe hematological toxicity, were less frequent in the P‐PEG group, which might have contributed to the favorable clinical outcomes in these patients. These data further support the prophylactic use of hematopoietic colony‐stimulating factors in patients with SCLC being treated with AMR.

However, this study had several limitations. First, it was a single‐center retrospective study with a small sample size. Second, the study included both refractory and sensitive relapses, which allowed for heterogeneity in terms of patient backgrounds. Studies on larger number of patients are needed to confirm the superior survival conferred by AMR with P‐PEG therapy for refractory and sensitive relapsed SCLC. Third, we could not assess the quality of life of patients and cost effectiveness of therapy. The reduction in severe hematologic toxicity, such as FN, could be related to improvements in the quality of life of patients and cost effectiveness, which should be evaluated in the future. In order to resolve the above three limitations, we will continue to accumulate and examine further cases.

In conclusion, AMR in combination with P‐PEG as second‐line chemotherapy for patients with SCLC reduced the incidence of FN without AMR dose adjustment and was associated with favorable tumor responses and survival outcomes compared to those observed with AMR monotherapy. P‐PEG should be considered for patients treated with AMR for SCLC, especially those with refractory relapsed SCLC.

## AUTHOR CONTRIBUTIONS

Conceptualization: Motoki Sekikawa and Haruyasu Murakami. Methodology: Motoki Sekikawa and Haruyasu Murakami. Project Administration: Haruyasu Murakami. Formal Analysis: Motoki Sekikawa. Writing—Original Draft: Motoki Sekikawa. Writing–Review & Editing: Meiko Morita, Kosei Doshita, Keita Miura, Hiroaki Kodama, Noboru Morikawa, Yuko Iida, Nobuaki Mamesaya, Haruki Kobayashi, Ryo Ko, Kazushige Wakuda, Akira Ono, Hirotsugu Kenmotsu, and Tateaki Naito. Supervision: Hirofumi Chiba and Toshiaki Takahashi. All authors have read and approved the final version of the manuscript.

## CONFLICT OF INTEREST STATEMENT

Motoki Sekikawa reports personal fees from Chugai pharma, outside the submitted work. Haruyasu Murakami reports grants from AstraZeneca, Chugai pharma, Takeda, Abbvie, Daiichi Sankyo, and IQvia and personal fees from AstraZeneca, Chugai pharma, Takeda, Abbvie, Daiichi Sankyo, MSD, Ono Pharmaceutical, Bristol‐Myers Squibb Japan, Pfizer, Novartis, Eli Lilly Japan, Taiho Pharmaceutical, Boehringer Ingelheim, Eisai, and Nippon Kayaku, outside the submitted work. Kosei Doshita reports personal fees from Eli Lilly Japan, AstraZeneca, and Chugai pharma, outside the submitted work. Keita Miura reports personal fees from Chugai pharma and Taiho Pharmaceutical, outside the submitted work. Hiroaki Kodama reports personal fees from Chugai pharma, AstraZeneca, and Boehringer Ingelheim, outside the submitted work. Nobuaki Mamesaya reports grants from Boehringer Ingelheim and personal fees from Chugai pharma, AstraZeneca, Boehringer Ingelheim, Taiho Pharmaceutical, MSD, and Ono Pharmaceutical, outside the submitted work. Haruki Kobayashi reports personal fees from Eli Lilly, Novartis, Taiho Pharmaceutical, Chugai pharma, AstraZeneca, Ono Pharmaceutical, and Bristol‐Myers Squibb Japan, outside the submitted work. Ryo Ko reports grants from MSD and AstraZeneca and personal fees from Taiho Pharmaceutical, Chugai Pharmaceutical, Eli Lilly, Boehringer Ingelheim, Pfizer, AstraZeneca, Ono Pharmaceutical, Daiichi Sankyo, and Takeda, MSD, outside the submitted work. Kazushige Wakuda reports grants from AstraZeneca, Chugai pharma, Novartis, Abbvie, AMGEN, Daiichi Sankyo, and Dizal Pharma and personal fees from Chugai pharma, AstraZeneca, Taiho Pharmaceutical, Boehringer Ingelheim, Eli Lilly, MSD, Ono Pharmaceutical, Daiichi Sankyo, Jassen Pharmaceutial, Takeda, and Nippon Kayaku, outside the submitted work. Akira Ono reports personal fees from Chugai pharma, AstraZeneca, and Ono Pharmaceutical, outside the submitted work. Hirotsugu Kenmotsu reports grants from Ono Pharmaceutical, Novartis, Eli Lilly, AstraZeneca, and Loxo Oncology and personal fees from AMGEN, AstraZeneca, Bayer, Boehringer Ingelheim, Bristol‐Myers Squibb, Chugai pharma, Daiichi Sankyo, Eli Lilly, Kyowa Hakko Kirin, Merck, MSD, Novartis, Ono Pharmaceutical, Pfizer, Taiho Pharmaceutical, and Takeda, outside the submitted work. Tateaki Naito reports grants from Otsuka Pharmaceutical and personal fees from Ono Pharmaceutical and HELSINN SA, outside the submitted work. Hirofumi Chiba reports personal fees from Boehringer Ingelheim, outside the submitted work. Toshiaki Takahashi reports grants from AstraZeneca, Chugai Pharma, Eli Lilly Japan, Ono Pharmaceutical, MSD, Pfizer, AMGEN, Boehringer Ingelheim, and Merck Biopharma and personal fees from AstraZeneca, Chugai Pharma, Eli Lilly Japan, Ono Pharmaceutical, MSD, Pfizer, Boehringer Ingelheim, Roche Diagnostics, and Takeda, Yakult, outside the submitted work. All other authors have nothing to disclose.

## Data Availability

The datasets used and/or analyzed during the current study are available from the corresponding author on reasonable request.
